# Supplementary material for: A Global Screen for Assembly State Changes of the Mitotic Proteome by SEC-SWATH-MS
Source: Cell Syst. 2020 Feb 26;10(2):133–155.e6. doi: 10.1016/j.cels.2020.01.001 (PMC7042714; doi:10.1016/j.cels.2020.01.001)

Q92620 | PRP16_HUMAN | DHX38 DDX38 KIAA0224 PRP16
Monomer MW [kDa]: 140.503 Monomer expected elution fraction: 37

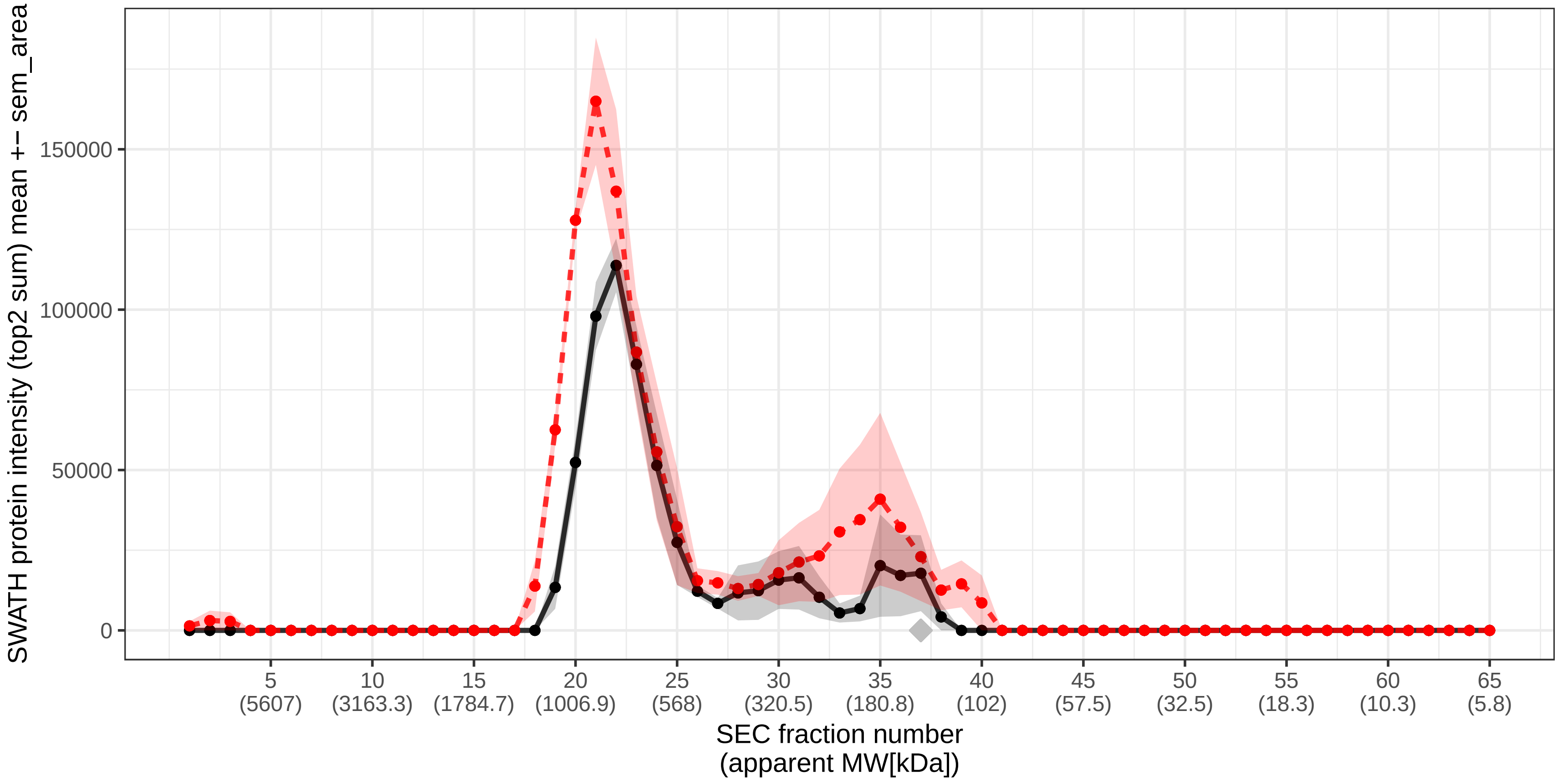

Supplement: Data S1. SEC-SWATH-MS Protein Chromatograms, Related to Figure 1 [file mmc6.zip › SECchrom_Q92620_PRP16_HUMAN_DHX38_DDX38_KIAA0224_PRP16.pdf]
